# Supplementary material for: The Influence of Competitive Level on Stretch-Shortening Cycle Function in Young Female Gymnasts
Source: Sports (Basel). 2022 Jul 6;10(7):107. doi: 10.3390/sports10070107 (PMC9323029; doi:10.3390/sports10070107)
Supplement: Supplementary file 1 [file sports-10-00107-s001.zip › sports-1634583-supplementary.pdf]

Table S1. Drop jump variables description

Variable	Units	Description
Jump height	m	The greatest vertical displacement of the centre of mass during the flight time. This was calculated using methods by Leard (Leard et al. 2007).
Ground contact time (CT)	s	The time interval of the ground contact of the first landing. This was established using the first data point greater than 15 N (i.e. initial ground contact) and the final data point that exceeded 15 N (take-off).
Reactive strength index (RSI)	Arbitrary units	The ratio between jump height (mm) and first ground contact time (ms).
Peak centre of mass displacement (ΔCOM)	m	The peak vertical displacement of the body's centre of mass during the first ground contact.
Spring-like correlation	Arbitrary units	The correlation between centre of mass displacement and absolute vertical force throughout the first ground contact.
Relative braking peak force	BW	The highest transient, visible force peak during the landing phase divided by bodyweight.
Time of landing peak force	%	Time interval between the first landing and landing peak as a percentage of total ground contact time.
Relative propulsive peak force	BW	The highest transient, visible force peak during the take-off phase divided by bodyweight.
Net impulse	(N.s)	Total impulse during the first ground contact minus bodyweight impulse.
Braking impulse	(N.s)	The sum of impulse between the first landing and the peak centre of mass displacement.
Propulsive impulse	(N.s)	The sum of impulse between the peak centre of mass displacement and take-off.
Braking duration	(s)	The time interval between the first landing and the peak centre of mass displacement.
Propulsive duration	(s)	The time interval between the peak centre of mass displacement and take-off.
Ratio of braking: propulsive impulse	Arbitrary units	Braking impulse divided by propulsive impulse.
